# Variation in *Anopheles* distribution and predictors of malaria infection risk across regions of Madagascar

**DOI:** 10.1186/s12936-020-03423-1

**Published:** 2020-09-29

**Authors:** Nicholas J. Arisco, Benjamin L. Rice, Luciano M. Tantely, Romain Girod, Gauthier N. Emile, Hervet J. Randriamady, Marcia C. Castro, Christopher D. Golden

**Affiliations:** 1 Department of Global Health and Population, Harvard T.H. Chan School of Public Health, 655 Huntington Avenue, Building 2, Room 329, Boston, MA 02115 USA; 2 Department of Organismic and Evolutionary Biology, Harvard University, Cambridge, USA; 3 Medical Entomology Unit, Institut Pasteur de Madagascar, Antananarivo, Madagascar; 4 Madagascar Health and Environmental Research (MAHERY), Antananarivo, Madagascar; 5 Department of Environmental Health, Harvard T.H. Chan School of Public Health, Boston, USA; 6 Department of Nutrition, Harvard T.H. Chan School of Public Health, Boston, USA

**Keywords:** Land use change, Planetary health, Disease ecology, Vector-borne disease, Malaria

## Abstract

**Background:**

Deforestation and land use change is widespread in Madagascar, altering local ecosystems and creating opportunities for disease vectors, such as the *Anopheles* mosquito, to proliferate and more easily reach vulnerable, rural populations. Knowledge of risk factors associated with malaria infections is growing globally, but these associations remain understudied across Madagascar’s diverse ecosystems experiencing rapid environmental change. This study aims to uncover socioeconomic, demographic, and ecological risk factors for malaria infection across regions through analysis of a large, cross-sectional dataset.

**Methods:**

The objectives were to assess (1) the ecological correlates of malaria vector breeding through larval surveys, and (2) the socioeconomic, demographic, and ecological risk factors for malaria infection in four ecologically distinct regions of rural Madagascar. Risk factors were determined using multilevel models for the four regions included in the study.

**Results:**

The presence of aquatic agriculture (both within and surrounding communities) is the strongest predictive factor of habitats containing *Anopheles* larvae across all regions. Ecological and socioeconomic risk factors for malaria infection vary dramatically across study regions and range in their complexity.

**Conclusions:**

Risk factors for malaria transmission differ dramatically across regions of Madagascar. These results may help stratifying current malaria control efforts in Madagascar beyond the scope of existing interventions.

## Background

Agricultural expansion and associated changes in land use, such as deforestation for food production, can create new microhabitats and alter the distribution or density of species, including mosquitoes. These changes can increase the number of suitable larval habitats for mosquito species, including *Anopheles* mosquitoes, the vectors for human malaria [1–4]. Changes in vector populations are mediated by local ecological factors, such as temperature, rainfall, relative humidity, the suitability of water habitats for mosquito larvae, and forest cover [5]. For example, deforestation in the Brazilian Amazon has increased suitable breeding habitats and conditions for certain *Anopheles* species, increasing vector density, while in the western Kenyan highlands, deforestation and changes to local ecology have lowered the survival time of certain *Anopheles* species [6, 7]. In addition to these ecological variables influencing population dynamics, biting rates, and malaria transmission potential of *Anopheles* vectors [7–10], human behaviour and resource access, here referred to as socioeconomic factors, also play key roles in mediating malaria risk. Examples of these socioeconomic factors include bed net use, health and housing infrastructure, baseline health status, and treatment/prevention access [11, 12]. These ecological and socioeconomic factors, and interactions among them and individual demographic factors such as age and gender, may also vary across geographic contexts. Thus, an individual’s risk for malaria infection results from a complex process involving socioeconomic, ecological, and demographic factors acting at the individual, household, and community levels, and likely vary regionally.

Though knowledge of risk factors for malaria infection is growing globally, only a limited number of studies explore how malaria risk factors vary across Madagascar’s diverse, rapidly transforming landscapes. Nationally, large-scale anthropogenic deforestation and land-use change are widespread [13–16]; 63% of Madagascar’s population of 26,262,368 lives in small farming communities in rural areas [17]. However, the distribution of land-use change rates, known malaria risk factors, and malaria prevalence patterns vary regionally in Madagascar [18–21]. Communities living in the central highlands, for example, experience unstable transmission and low prevalence coupled with a history of intense forest clearance and actively cultivated landscapes [16, 22]. In Madagascar’s east and west coasts, deforestation rates are increasing. However, communities living in western dry forests experience more seasonal transmission while those living in eastern moist forests experience more consistent transmission throughout the year [23].

In rural Madagascar, humans live at the interface of their communities and their surrounding environments, spending substantial time in adjacent forests and agricultural fields. These interactions predicate many facets of population health, including nutrition, exposure to infections, and sanitation practices [24–26]. However, little information exists on the extent that these exposures alter malaria risk across ecologically distinct regions of Madagascar. Additionally, the natural and modified habitats surrounding human communities differ greatly between ecologically distinct regions of Madagascar [27]. In eastern humid tropical forest, *Anopheles* mosquitoes are more abundant in agricultural land and village environments than surrounding forests, indicating forest clearance as a possible driver of local malaria transmission [28]. A cross-sectional study of communities in southeast Madagascar demonstrated that bed net use protected against malaria, while rural individuals in lower socioeconomic brackets, between 6 and 14 years of age, were at higher risk of infection [23, 29]. However, they did not collect information on malaria vector distribution, nor did they analyse these patterns in other regions.

Additionally, *Anopheles* species have varying vectorial capacities and larval habitat preferences [30–32]. However, little is known about the extent to which vector populations within and around local communities vary and how they respond to environmental change. For example, it has been hypothesized that as local ecology changes, opportunistic vectors move into places previously uninhabited, altering the profile of malaria risk across space [33]. However, understanding how the distribution of malaria risk has or will differ due to land use change is difficult given insufficient data on current distributions of malaria vector communities in rural communities. To this end, malaria risk factors and local vector ecology were characterized in this study among rural communities in multiple ecologically distinct regions of Madagascar.

A recent, large-scale cross-sectional survey showed marked heterogeneity in malaria prevalence between ecologically distinct regions and communities within regions in Madagascar (Rice et al. pers. commun.). In particular, the prevalence in some rural communities in southeast and southwest Madagascar was substantially higher than national reporting. Likewise, Kang et al. estimated a fourfold increase in the proportion of areas in Madagascar experiencing high transmission has occurred since 2011 [34]. This motivates the identification of risk factors associated with malaria infection in these areas to implement more efficient control methods.

The linkages between Madagascar’s unique ecologies, the distribution of malaria vectors, and malaria outcomes in human populations are unclear. As such, this study intends to identify (1) the ecological correlates of malaria vector larval presence through surveys of larval *Anopheles* mosquitoes within communities, and (2) the key socioeconomic, demographic, and ecological factors associated with increased odds of malaria infection in four ecologically distinct regions of rural Madagascar.

## Methods

### Site selection and population description

**Fig. 1:**
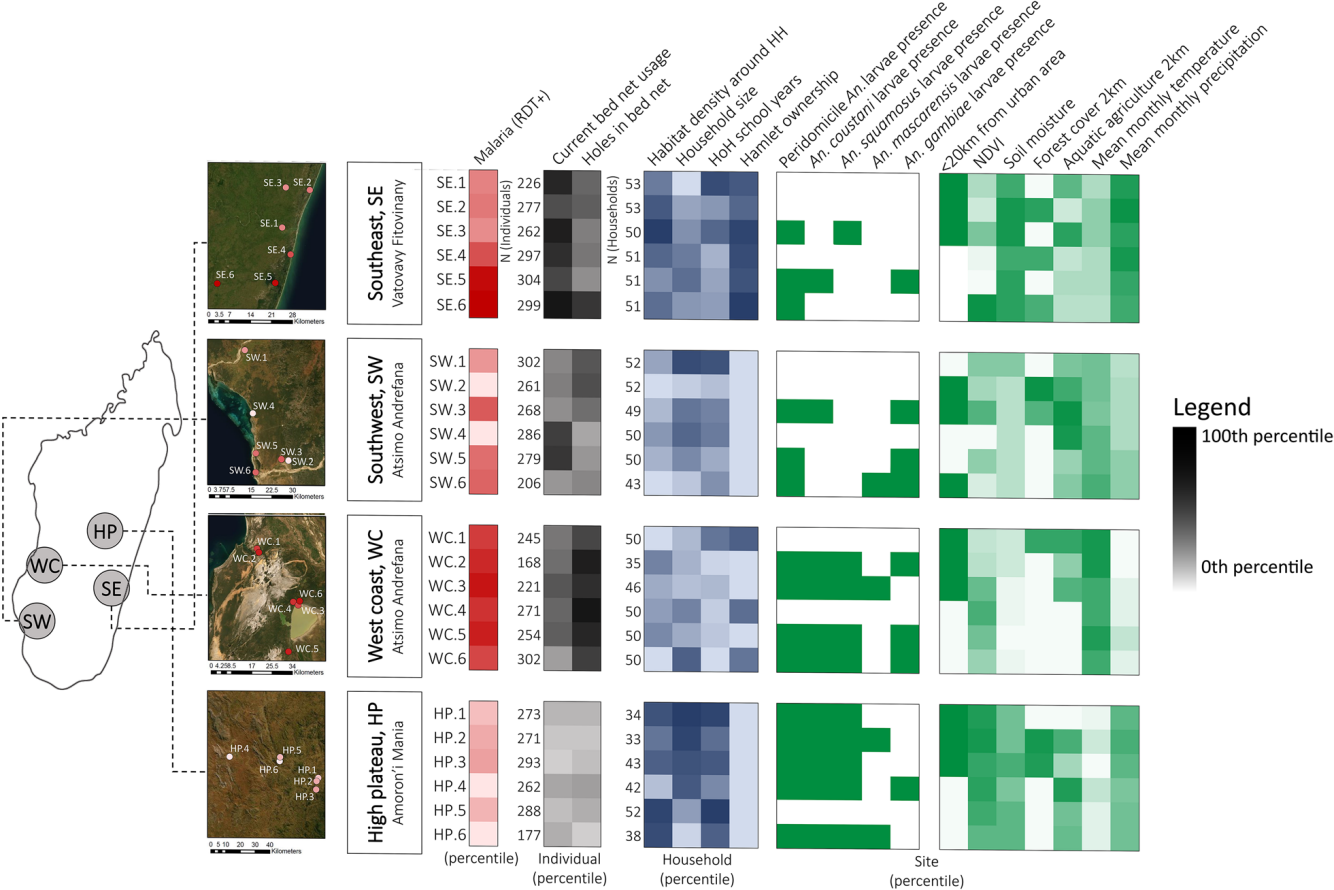
Heatmap of individual, household, and site-level predictors of the outcome: the proportion of the population with malaria (RDT +) by site. Darker colors signify higher percentiles, lighter colors signify lower percentiles. Satellite imagery obtained from SPOT6 and SPOT7 satellites. Site marker color corresponds to malaria prevalence in each site

Malaria outcome, socioeconomic, and demographic data were obtained from a cross-sectional study of 5602 participants across 24 communities in four regions of Madagascar (Fig. 1) [35]. Larval vector surveys and subsequent analyses were conducted in these four study regions. The study by Rice et al. (Rice et al. pers. commun.) included data from a second sub-region of the east coast (their NE region) but malaria prevalence estimates and study design differed and thus this region is omitted here. Each region will be referred to by its geographical location throughout the paper; these are listed here along with their corresponding administrative districts: southeast, SE = Vatovavy Fitovinany: Mananjary district; southwest, SW = Atsimo Andrefana: Toliara II district; west coast, WC = Atsimo Andrefana: Morombe district; and high plateau, HP = Amoron’i Mania: Ambositra, Ambatofinandrahana, and Fandriana districts.

Sampling regions were selected to represent the different typologies of malaria transmission patterns in Madagascar, ranging from consistent, endemic transmission in the east, to episodic, epidemic transmission in the central highlands [22, 29]. These zones correspond to Madagascar’s distinct ecological regions, with differing precipitation and vegetation patterns and include humid forest, grasslands, dry spiny forest-thicket, and others [36]. The landscapes in these regions are a mosaic of original, natural vegetation and human modified areas where secondary forest, cultivated land, and pasture dominate. Ecotype descriptions were adapted from Goodman et al. and Moat & Smith [27, 37]. The SE region falls under the ecotype humid forest, characterized by degraded humid forest, wooded grassland-brushland mosaic, lowland moist evergreen forest, secondary forest, and secondary grasslands and experiences year-long, endemic malaria transmission. The SW region falls under the ecotype dry spiny forest and is characterized by dry spiny forest-thicket, degraded dry spiny forest-thicket, mangroves, secondary forest, and secondary grasslands and experiences endemic, but seasonal malaria transmission. The WC region falls under the ecotype dry deciduous forest and is characterized as dry forest, dry spiny forest-thicket, mangroves, secondary forest, and secondary grasslands and experiences endemic, but seasonal malaria transmission. Finally, the HP region ecotype is a wooded grassland-bushland mosaic, characterized by plateau grassland-bushland mosaic, secondary forest, and secondary grasslands and experiences seasonal, episodic malaria transmission. The SW and WC regions denoted in this study are both part of the Atsimo Andrefana region of Madagascar, but these regions are nearly 200 km from each other and are characterized by distinct climatic and environmental profiles, thus providing us motivation to consider them as separate.

In this study, communities are defined as clusters of households (the typical rural settlements in these areas, *tanàna* or *tanàna kely*) and the adjacent peri-domicile areas. Sites are defined as communities and the surrounding mosaic of cultivated land, uncultivated human-modified land, and undisturbed land. In all communities, sampling was performed by randomly selecting ~ 50 households with two selection criteria: (1) a child five years of age or younger; and (2) a woman of reproductive age (15–49 years) (See [35] for more details on enrollment). Six communities were sampled in each region, three more proximate to an urban area (defined as < 20 km) and three more distant (defined as > 20 km) from an urban area. 33–53 households (168–304 individuals) were sampled per site and 242–309 households (1461–1665 individuals) were sampled per region, though only complete cases were used for the analysis.

### Malaria outcome and associated socioeconomic and demographic variables

Health and survey data were collected at the individual and household level [35]. In total, 10 of these variables were used (see Additional file 1: Table S1). One key variable included, hamlet ownership, is defined as a household owning a separate home away from the community proper that serves as a base for easier access to agricultural settings. Age of individuals included in the analysis was divided into four groups: under 5 years (reference category), 5–14 years, 15–64 years, and 65 + years. This breakdown was motivated by the literature [37–39] that often show distinct differences in malaria prevalence across these age groups. Many data sources specifically sample children under 5, because they often bear the highest malaria mortality [40]. Therefore, we use this age group as reference to aid in comparison of estimates here with other studies. Rapid diagnostic tests (RDTs) were used to diagnose malaria (SD Bioline Malaria Ag P.f/Pan RDT). False negativity and the contribution of infections by other species such as *Plasmodium vivax* are known limitations of RDT based surveys [41], however, RDT positivity is justifiably a reliable proxy for malaria infection as previous studies using these RDTs in Madagascar found high agreement when comparing between RDT results and molecular detection via PCR (over 87% sensitivity and specificity) [41–43]. Regarding *Plasmodium* species, over 96% of malaria cases in Madagascar are due to *Plasmodium falciparum* [40, 44] and in previous molecular confirmation of RDT positive cases from the east coast of Madagascar, over 98% were *P. falciparum* infections [42]. A total of 776 individuals were positive by RDT for malaria, varying from 6 to 381 individuals per region.

### Ecological variables and Anopheles larval sampling

To complement the data described above, ecological surveys were conducted in the 24 study communities to determine ecological risk factors for malaria transmission between the months of May 2017–August 2017. Larval surveys were conducted during this period to sample vectors during, or as close to peak transmission season as possible, in all zones. Seasonal transmission dynamics exist in these regions, but we attempt to circumvent challenges presented by them through overlapping sampling times. The overlap between larval sampling and RDT testing in the SE and SW differed by up to five and up to three months, respectively. The peak transmission period in these regions has been found to be from January to May or June [20], so all larvae were collected during the peak transmission season for each region. Thus, it is not expcted that the mismatched sampling times would bias the results (For further seasonal analysis, see Additional file 2: Table S2).

All potential and positive larval habitats were mapped with a Garmin Oregon 550t prior to larval collection. All habitats within a 25 m radius of households or the community perimeter were geocoded, and mosquito larvae were sampled from each. Transect mapping was conducted to identify larval habitats and species composition of the local ecotype of each research site (all mosquito sampling methods described in detail in Additional file 2).

All larvae were sorted by genus and instar prior to identification by morphological examination. *Anopheles gambiae* is a complex of species represented by *Anopheles gambiae* sensu stricto*, Anopheles arabiensis*, and *Anopheles merus* in Madagascar. These three species are indistinguishable by morphology, and necessitate molecular identification [45]. As such, larval specimens identified by morphology will be collectively referred to as in *An. gambiae* complex. All third/fourth instar larvae were identified morphologically to the lowest possible taxonomic level using the taxonomic key presented in Grjebine [46] at Institut Pasteur Madagascar. Some *Anopheles* larvae were unidentifiable to species because they were damaged in the sampling process which removed features necessary to distinguish species.

In Madagascar, aquatic forms of agriculture are used for farming predominantly rice, but this method is also used for growing other types of plants, and some spaces remain empty while still pooling water. As such, aquatic agriculture is defined as a combination of all forms of agriculture reliant on a flooded field. Aquatic agriculture cover was determined through ground truthing and remote sensing. Distance from the household waypoint to the edge of the nearest aquatic agriculture was calculated. For each household, the percentage area that was aquatic agriculture within a 1 km buffer was calculated. Finally, all possible mosquito breeding habitats within a 25 m buffer were enumerated around all households. All spatial variables were calculated using ArcMap and all satellite imagery was purchased from the AIRBUS Spot 6 and 7 Satellites at 1.5 m spatial resolution.

### Statistical analyses

To assess ecological correlates of the odds of mosquito larvae presence in sampled habitats, we modelled five outcomes using simple logistic regression: (A) Presence/absence of *Anopheles* larvae, (B) Presence/absence of *An. gambiae* complex larvae, (C) Presence/absence of *Anopheles mascarensis* larvae, (D) Presence/absence of *Anopheles coustani* larvae, and (E) Presence/absence of *Anopheles squamosus* larvae. For model (A), data were restricted to habitats containing any genus of mosquito larvae. For models (B)–(E), habitats included in the model were only those with *Anopheles* mosquito larvae present, which was a subset of all habitats. Predictors included in model (A) were the Normalized Difference Vegetation Index (NDVI, proxy of green vegetation cover calculated via remote sensing of satellite imagery; value calculations apply to the month in which larval sampling took place) at each habitat [47], habitat type (aquatic agriculture, ponds, containers), percent of forest cover in a 1 km radius around the centroid of each site [10], soil moisture of each site [48], temperature of each site (mean monthly [49]), and percent of aquatic agriculture in a 1 km radius around the centroid of each site [50] (ESM 1 lists variables/sources/tests for correlation of variables). Variables included in models (B)–(E) were identical to model (A), with indicator variables for the presence of each *Anopheles* species. These data were analysed using logistic mixed-effects models with random effects at the site level.

To assess the ecological, socioeconomic, and demographic correlates for the odds of malaria infection across the four study regions, four multilevel logistic regression models were employed, one for each region, with random effects for households nested within communities. The outcome of interest was malaria infection status, as diagnosed by RDT. Separate models were employed for each region due to the high variation in malaria prevalence (Rice et al. pers. commun.) and distinct ecology of each region [27].

The SE and WC regions began with the same full model, and the SW and HP began with slightly modified models. The difference in models was due to household hamlet ownership in SW and HP having none or limited heterogeneity. Incomplete cases were excluded from analyses (n = 214). To test for bias induced by complete case analysis, correlations were assessed between remaining individuals and the outcome of interest. The correlation estimates from these data were within the confidence bounds of the correlation estimates from the full dataset, and thus no significant change in estimates after exclusion of individuals with missing data. All statistical analyses were conducted in R v3.5.1 with the lmerTest package [51, 52].

## Results

A total of 4661 individuals were included in the study after exclusion of incomplete cases (Fig. 1). 61.9% of individuals surveyed reported using bed nets at the time of the survey. Bed net coverage during sampling was highest in the SE (95.1%), then SW (80.2%), WC (75.2%), and HP (12.3%). Of those using bed nets, 35.9% stated that their bed net had holes. 41.4% of non-bed net users were children under 18 years, and 52.7% were female.

**Fig. 2:**
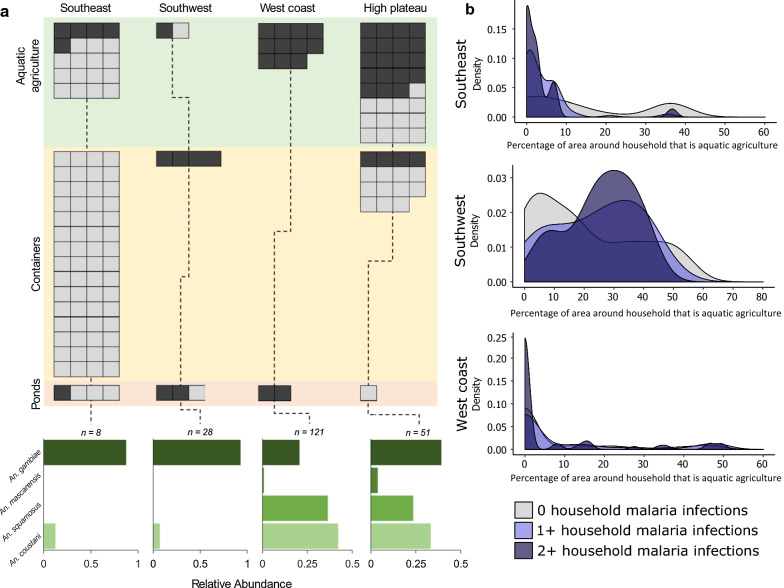
Larval mosquito species information. **a**: Larval habitats found near households in rural Madagascar, separated by region and habitat type. Dark grey boxes indicate habitats with *Anopheles*, while light grey boxes indicate habitats with other genera of mosquitoes. *Anopheles* species abundance in each region is displayed in the bar charts below. **b** Distribution of the amount of aquatic agriculture surrounding households (within a 500-m radius) with 0 (grey), 1 + (light blue), or 2 + (dark blue) malaria infections. The HP region was not included due to too few individuals with malaria

From larval sampling, a total of 661 *Anopheles* larvae were identified, of which 448 were third/fourth instar and identifiable to the species/species complex level. *Anopheles* larvae were present in 16 of 24 study sites and in every region. The most common species of *Anopheles* identified was *An. gambiae* complex (43.8% of all larvae), followed by *An. coustani*, *An. squamosus,* and *An. mascarensis* (Fig. 2). 70% of *Anopheles* species were collected from aquatic agriculture systems, and all species were found most commonly in these systems. *Anopheles coustani* was dominant in the SE, HP, and WC, while *An. gambiae* was dominant in the Southwest. Aquatic agriculture covered 0% to 56.4% of the area in a 1 km radius around a household. The average number of peridomicile habitats varied by region (from 0.24 to 2.82), with larger numbers on average existing in HP (2.82 habitats) and SE (2.71 habitats) compared to SW (0.24 habitats) and WC (0.37 habitats).

### Ecological correlates of increased/decreased odds of *Anopheles* larvae presence

The variables in larval models B-E predicting presence/absence of *An. gambiae* complex, *An. mascarensis*, *An. coustani*, and *An. squamosus* larvae were not statistically significant or data were too sparse for proper model fitting. Results from these models lacked power and are not reported. In larval model (A), however, two variables were statistically significant (P < 0.05), which predicted presence/absence of *Anopheles* larvae in each habitat, and were if the habitat was aquatic agriculture and higher average soil moisture levels (Additional file 2: Table S1). Since no other variable was statistically significant, risk factors including socioeconomic, demographic, and further ecological variables were explored as potential risk factors.

### Ecological and socioeconomic correlates for the increased/decreased odds of malaria infection

#### Southeast (Vatovavy Fitovinany, SE)

Six variables were statistically significant (P < 0.05) for SE Madagascar and were associated with an individual’s increased odds of having malaria (Table 1). These variables, from most influential in increasing the odds of malaria infection in an individual to the least influential, were: individuals living in more remote communities (> 20 km from an urban area); the presence of *Anopheles* larvae within communities; each of the other age groups, 5 to 15, 15 to 64, and 65 + ; if the individual lived in a household that owns a hamlet; and if the individual was a male.

**Table 1:** Generalized linear mixed-effects model output results. Random effects = household nested within site

Results								
	Odds of an individual being RDT + for *P. falciparum* compared to RDT-(95%CI)							
	Vatovavy Fitovinany, SE		Atsimo Andrefana (Southwest, SW)		Atsimo Andrefana (West coast, WC)		Amoron’i Mania, HP	
Site								
*Anopheles* mosquitoes present in peridomicile habitat	4.39^**^	(2.92, 6.59)	12.70^**^	(3.33, 48.43)	0.67	(0.38, 1.20)	N/A	N/A
> 20 km from urban center	10.00^**^	(5.56, 16.67)	0.69	(0.29, 1.61)	0.47^*^	(0.25, 0.87)	1.03	(0.03, 33.33)
Household								
1-unit increase in count of peridomicile larval habitats	0.95	(0.87, 1.03)	0.97	(0.39, 2.42)	0.91	(0.72, 1.13)	0.73	(0.43, 1.24)
Distance from household to nearest aquatic agriculture (km)	1.25	(0.80, 1.93)	2.51	(0.24, 26.17)	2.05	(0.85, 4.93)	1.08	(0.10, 11.32)
% of land used as aquatic agriculture 1 km around household	1.01	(0.96, 1.01)	1.10	(0.99, 1.24)	1.01	(1.00, 1.03)	0.89	(0.76, 1.05)
Head of household high school education	1.20	(0.72, 1.48)	0.85	(0.44, 1.64)	0.54^**^	(0.37, 0.81)	0.28	(0.03, 2.32)
Household has income source	1.27	(0.82, 2.08)	0.72	(0.32, 1.64)	1.09	(0.72, 1.63)	0.40	(0.04, 4.44)
Household owns a hamlet	1.20^*^	(1.01, 2.04)	N/A	N/A	0.13^*^	(0.02, 0.96)	N/A	N/A
Individual								
Currently using bed net	0.74	(0.37, 1.46)	0.79	(0.39, 1.61)	0.66^*^	(0.44, 0.97)	00.33	(0.00, 99.79)
Bed net used has holes	1.00	(0.68, 1.46)	0.41	(0.13, 1.26)	0.74	(0.53, 1.03)	69.01	(0.18, 26196.97)
Ages 5 to 14	1.58^**^	(4.70, 28.57)	1.04E + 10	(1.55E−128,6.91E + 147)	32.22^**^	(7.68, 135.20)	1.07E + 09	N/A
Ages 15 to 64	1.57^**^	(1.86, 10.94)	2.19E + 09	(3.27E−129, 1.46E + 147)	15.46^**^	(3.70, 64.65)	7.18E + 08	N/A
Ages 65 +	1.63^**^	(5.30, 36.39)	9.93E + 09	(1.49E−128, 6.62E + 147)	44.07^**^	(10.17, 190.86)	2.05E + 09	N/A
Male	1.17^**^	(1.27, 2.37)	1.02	(0.54, 1.91)	1.67^**^	(1.28, 2.17)	0.30	(0.03, 2.93
Observations	1299		906		1252		1205	
Random Effects Variance (SD)								
Site	0.82 (0.90)		0.99 (0.99)		0.71 (0.84)		0.79 (0.89)	
Household	1.95E−13 (4.41E−7)		1.06E−13 (3.26E−7)		6.86E−14 (2.62E−7)		3.16E14 (1.78E−7)	

Estimates are odds ratios. N/A variables signify those that were not included in models

^*^p < 0.05, ^**^p < 0.01

### Southwest (Atsimo Andrefana: Toliara II District, SW)

The malaria prevalence in this region was 5.7% overall (range 0–16.4% by site), and therefore limited heterogeneity exists in the outcome variable of interest. As such, some estimates have wide confidence limits and should be interpreted with caution. One variable was statistically significant (P < 0.05) for SW Madagascar and was associated with an individual’s increased odds of having malaria: presence of *Anopheles* larvae in a community.

#### Central West Coast (Atsimo Andrefana: Morombe District, WC)

Eight variables were statistically significant (P < 0.05) for WC Madagascar, four of which were associated with an individual’s increased odds of having malaria and four of which was associated with an individual’s decreased odds of malaria (i.e. a protective effect). Variables associated with an increased odds of having malaria, from most to least influential, were: each of the age groups, 5 to 15, 15 to 64, and 65; and if the individual was male. Variables associated with a decreased odds of having malaria, from most influential to least influential, were: if the individual lives in a household that owns a hamlet (opposite direction as SE estimate); if the individual lives > 20 km from an urban area (opposite direction as SE estimate); if the individual lived in a household with a high school-educated head of household; and if the individual uses bed net.

#### High Plateau (Amoron’i Mania, HP)

The malaria prevalence in this region was 0.4% overall (range 0–0.97% by site), and therefore limited heterogeneity exists in the outcome variable of interest. Estimates for this region are unstable (random effects variation: 3.16 × 10^14^) and should be interpreted with caution. No variables included in this region’s model were statistically significant.

## Discussion

This is a comprehensive study on risk factors for *Anopheles* larvae presence and malaria infection across regions of Madagascar with distinct ecologies, climates, and transmission patterns. The goals of this study were two-fold: (1) to understand potential ecological drivers of *Anopheles* mosquito presence in peri-domicile habitats across rural Madagascar and (2) to identify socioeconomic, demographic, ecological, and geographic risk factors associated with malaria infection. *Anopheles* larvae were more commonly found in peri-domicile aquatic agriculture as compared to other types of peri-domicile habitat as well as sites with a higher soil moisture level on average, but that no other ecological variables predicted peri-domicile *Anopheles* larvae presence. Differences in risk factors were found between study regions, with some regions demonstrating more complex suites of risk factors than others. For example, *Anopheles* presence in peri-domicile habitat predicted an increased risk of malaria in the SE and SW but was not in the WC. Current vector control efforts in Madagascar target adult mosquitoes, however, the data suggest that locally managed control of peri-domicile larval sources could be an effective, novel intervention in SE and SW Madagascar. Furthermore, agricultural practices are a clear driver of the presence of *Anopheles* positive larval habitats across all regions, and thus larvicide approaches and inclusion of alternative agricultural practices may serve as an effective means to reduce human contact with vectors.

This study has limitations, namely diagnosis by RDT as discussed above, and the scope of larval sampling. More accurate testing methods, like PCR of malaria parasites, may reduce bias introduced by RDT sensitivity/specificity. Additionally, most sampling of larvae was limited to habitats within 25 m of households. Malaria prevalence in some communities with few peridomicile habitats was high compared to regional averages (17% in site SW.3, few peridomicile habitats but bordered by valley of rice fields ~ 0.5 km away). *Anopheles gambiae* adults have been observed to have maximum flight distances of 0.2 to 6.4 km [53]. Larval sources more distant from households may be responsible for risk but little evidence exists [54]. In support, in the SE, hamlet ownership increased malaria risk. Household proximity to larval sources did not predict malaria risk in the WC region, which had the highest mean prevalence. As such, vector management may need to account for more distant larval sources.

**Fig. 3:**
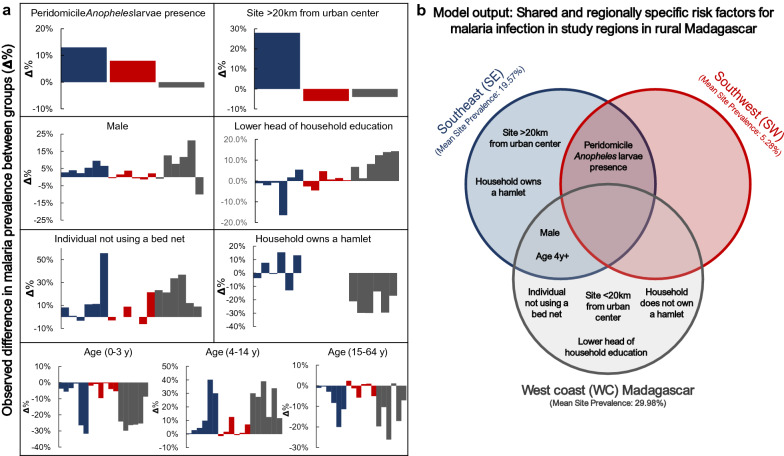
**a** The observed difference in malaria prevalence between groups. Variables are at the site or regional levels, such that bars represent the regional average or the site average depending on how they were coded for the models. Bars > 0% demonstrate increased risk, bars < 0% demonstrate decreased risk. Colours correspond to their respective Venn diagram colors in (**b**). **b** Venn diagram demonstrating statistically significant predictors for increased malaria risk in the multilevel models employed for each region

The four regions included in this study vary in their ecology and malaria transmission patterns, and statistical analyses employed here demonstrate different risk factors associated with malaria infection in each region at the site-, household-, and individual-level (Table 1, Fig. 3). According to the National Malaria Indicator Survey conducted between May and July 2016, countrywide prevalence of malaria infection in children under 5 is about 6%, and regional prevalence ranges from 1 to 15% [55]. Though study data are not nationally representative, site prevalence estimates indicate high heterogeneity within regions, and that regional/national statistics should be interpreted cautiously. These data demonstrate high variation between sites and alarmingly high prevalence in the WC and SE. In the WC, drivers of increased malaria risk were higher urbanicity, living in a household with a less-educated head, and not using a bed net. In the SE, drivers of increased malaria were the presence of vector larvae in peridomicile habitat and lower urbanicity. Bed net usage significantly reduced an individual’s risk for malaria in the WC. However, coverage in the WC was significantly lower than coverage in the SE and SW (χ^2^-test for independence, P < 0.001).

From an ecological perspective, in the SE and the SW, the presence of *Anopheles* larvae was associated with an increased risk of malaria infection. The SW region is dry, with average annual rainfall below 400 mm [56]. This value is low compared to other study regions and accompanies a shift in dominant agriculture from flooded rice farming to dry-land farming. One out of the six SW sites had some form of aquatic agriculture, though *Anopheles* larvae were found in three of the six surveyed sites. In sites without rice fields, larvae were found in small, man-made ponds. Additionally, in both sites, children under 5 had malaria infections, indicative of local transmission as children are less likely to travel outside the community [12]. *Anopheles* larvae presence in these two sites was confined to a few small, easily identifiable locations that may be driving sustained transmission at the community level. In the SW region, application of larvicide to these few, identifiable *Anopheles* habitats may reduce malaria transmission burden significantly. Since water in these habitats is used for human consumption, a non-toxic larvicide is recommended [57].

*Anopheles* species composition varied by site, region, habitat preference, and relative abundance and their ecology is important to consider when comparing study regions (Fig. 2). Within the *An. gambiae* species complex, *An. gambiae* and *An. arabiensis* oviposit in freshwater sites, while *Anopheles merus* targets brackish habitats [45]. These three species are known as major malaria vectors in Madagascar [45]. *Anopheles mascarensis* is a secondary major vector in Madagascar, and breeds in fresh and brackish habitats [58]. Recent findings showed *An. coustani* was a competent malaria vector in Madagascar for both *P. vivax* and *P. falciparum* [59]. *Anopheles coustani* oviposit in freshwater and brackish habitats [30, 46]. *Anopheles squamosus* is suspected to mainly transmit malaria in the HP region due to high abundance [58]. The results of modelling of larval habitat preference align with other studies in Madagascar. Marrama et al. showed rice fields had the highest percentage of mosquito larvae and provide half of all positive larval breeding habitats [60]. Zohdy et al. demonstrated that *Anopheles* species are the dominant mosquitoes in agricultural and village settings, and are the minority in forest settings [28].

The relationship between rurality and malaria risk demonstrates regional heterogeneity. In the SE region, which is characterized by year long malaria transmission and tropical forest, individuals living closer to urban areas were at lower risk of malaria infection. This may reflect a relationship between less remote sites and more distant *Anopheles* breeding habitats, more accessible healthcare, cash income from markets, and wage labour access which can support prevention or treatment [61]. The opposite was found in the WC region, which is comparatively much drier and experiences seasonal malaria transmission. Here, the urban center is coastal and near a gradually widening river estuary area. Communities near this centre sit closer to slow-moving water, some of which has been diverted to flooding rice fields. Increases in wetland areas around communities proximate to the coastal urban center may increase *Anopheles* breeding habitats near less remote sites. *Anopheles mascarensis* and *An.coustani* have been found in brackish water, and both were present five of six sites in the WC. In the SW and HP, rurality had no significant effect on malaria prevalence. Of note, more remote and less remote sites generally had better road access in the SW and HP than in the SE and WC. As such, living in more remote versus less remote sites in the SW and HP may not be as different in occupational, and accessibility factors as in the SE and WC.

Hamlet ownership may be a risk factor that bridges the gap between socioeconomic and ecological factors in study regions. In the SE region, individuals were at higher risk of having malaria if they lived in a household owning a hamlet. Individuals in hamlets may be at higher risk of malaria infection because hamlets may be in locations with higher vector concentrations (i.e., near flooded rice paddies), have less protective household structures, or have fewer bed nets. Households owning hamlets may also be most dependent on seasonal agriculture, which may bring people into closer, more frequent contact with *Anopheles*-dense habitats. In contrast, households owning hamlets in the WC were protected against malaria infection, which may be explained by local ecology. In both regions, hamlets are located away from communities and other hamlets, but in the SE region, they are located in environments rife with mosquito habitats. Hamlets in the WC are in drier habitats where mosquito density may be lower than the center of communities, often located near water sources. These study findings indicate the need for future studies on vector exposure risk for hamlet owners and agricultural activity in the SE and WC of Madagascar.

From a socioeconomic perspective, in the WC, heads of households with a high school education or higher imparted lower malaria infection risk for all household members. Other studies demonstrate that individuals living in households with more educated household leaders have improved awareness of family health [62]. The observed association in the WC may indicate the need to increase awareness of symptoms and prevention mechanisms, such as bed net use. In the SE and WC regions, individuals 5 + years old and males were more susceptible to malaria infection compared to women and children under five. Women and children under five may spend more time nearer to households where transmission is less severe. Furthermore, individuals not using a bed net were not at different risk than individuals using a bed net. However, bed net use in the SE was 95.1%, so the sample size of those not using bed nets was limited. These relationships show that transmission is likely occurring outside households/community bounds. Bed nets remain effective in reducing malaria burden overall, but additional control measures may be needed in these areas to reduce transmission, such as better testing and treatment for high-risk individuals in the SE. Contrarily, bed net use in the WC protected against malaria infection. This association demonstrates that transmission of malaria may occur in households and communities proper. Therefore, distribution of bed nets in the WC region to ensure universal coverage may be an effective control strategy.

## Conclusion

Major risk factors for odds of malaria infection vary in their importance and directional affect across ecologically distinct regions of Madagascar. The results of this study demonstrate the need for spatially targeted malaria control strategies in Madagascar. Each geographical context should be considered independently prior to employing interventions. Malaria stratification, the process of classifying regions based on malaria risk to aid in resource distribution, has existed since the 1940s [63]. However, modern techniques like remote sensing, coupled with a growing case study literature, have amplified the effectiveness of this stratification [64, 65]. Though the national malaria control program of Madagascar stratifies by broad geographical zones, more granular stratification of control measures is needed. These results help clarify risk factors to augment and tailor current malaria control programmes according to socioeconomics, ecology, and geography. These data suggest the potential merits of sub-national, sub-regional stratification of malaria control efforts, specifically: (i) applying larvicide to peri-domicile habitats in the SE and SW, (ii) additional effort in distributing bed nets and prevention education in all regions but most heavily in the WC, and (iii) targeting all family members, but most acutely males over the age of 5, for active case detection in the SE and WC.

## Supplementary information

**Additional file 1.** Summary of variables used and their sources.

**Additional file 2.** In-depth larval sampling protocol, larval model results, correlation tests of variables included in region-specific models, and analysis of seasonality.

## Data Availability

Data and code for all models in this manuscript can be accessed here: https://figshare.com/projects/Variation_in_larval_ecology_and_predictors_of_malaria_risk_across_regions_of_Madagascar/78159.

## Abbreviations

SE: Southeast, Vatovavy Fitovinany: Mananjary district
SW: Southwest, Atsimo Andrefana: Toliara II district
WC: West coast, Atsimo Andrefana: Morombe district
HP: High plateau, Amoron’i Mania: Ambositra, Ambatofinandrahana, and Fandriana districts
RDT: Rapid diagnostic test
