# Supplementary material for: Variation in Anopheles distribution and predictors of malaria infection risk across regions of Madagascar
Source: Malar J. 2020 Sep 29;19:348. doi: 10.1186/s12936-020-03423-1 (PMC7526177; doi:10.1186/s12936-020-03423-1)
Supplement: Supplementary file 2 — Additional file 2. In-depth larval sampling protocol, larval model results, correlation tests of variables included in region-specific models, and analysis of seasonality. [file 12936_2020_3423_MOESM2_ESM.docx]

Mosquito larvae sampling protocol

Habitats were located by conducting a systematic search of the community area working in a grid system. All habitats within a 25m radius of households or the community perimeter were geocoded. Mosquito larvae were sampled from habitats, with methods dictated by habitat depth: habitats of depth >13cm were sampled for larvae with a standard BioQuip 350mL extendable mosquito dipper (*BQ Dipper with Extendable Handle, Item #1132BQH*) using the “complete submersion” method presented by O’Malley (≥2 samples taken per habitat) (1); habitats too narrow or shallow to be sampled via dipper (e.g., tree holes) were sampled using a 1oz BioQuip Aquatic Pipette (*BQ Aquatic Pipette 1 oz, Item #4776*). Habitats that were >2m in length in any direction were sampled at ≥4 equally spaced points along the edges and as close to the center as possible. Habitats that were <2m in any direction for which a dipper was used were sampled as close as possible to the center of the habitat. All mosquito larvae were sorted by placing entire samples into white trays and pipetting all third/fourth instar larvae into 95% ethanol for preservation. For samples with >100 larvae, a random sample of up to 50 third and fourth instar larvae were collected from the pooled samples.

We conducted transect mapping to identify larval habitats and species composition of the local ecology of each research site. Two 100m transects were mapped within or around each study community. Locations were chosen based on one area that was undisturbed and most representative of local ecology (e.g., dry forest, spiny thicket, etc.) and one area that represented the dominant human-altered land-use type (often agriculture). Undisturbed areas were usually on the periphery of sites (within 0.5 km of the center point of the community). We geocoded all mosquito suitable aquatic habitats along the transect and 5m to each side of the transect line. All distinct land-use types, such as agricultural fields, undisturbed areas, secondary forest, fallow land, etc., along the transect line were geocoded. When transecting along rice fields and other large habitats, a mosquito dipper was used to sample for larvae every 1 meter along the edge of the habitat. All larvae were sampled, sorted, identified and preserved in the same manner as described above.

References

1. O'MALLEY C. Seven ways to a successful dipping career. Wing Beats. 1995;6:23-4.

**Table 1**

Results from larval logistic regression model (A)

|  |  |  |  |  |  |
| --- | --- | --- | --- | --- | --- |
| Odds of larvae in positive sampling habitat being *Anopheles* species | | | | | |
| **Variable** | | **Odds Ratio** | | **Lower 95%CI** | **Upper 95%CI** |
| **Habitat Level Variables** | | | | |  |
| Habitat Type (Ref = Containers) | |  | |  |  |
| Aquatic agriculture | | | 6.26* | 2.73 | 9.80 |
| Pond | | | 2.02 | -4.35 | 8.39 |
| NDVI | | | 1.86 | -7.97 | 11.70 |
| **Site Level Variables** | | | | |  |
| Percent Forest Cover 2km Radius | | | 1.08 | -1.12 | 3.28 |
| Average soil moisture | | | 0.00 | -652.81 | 652.81 |
| Log(Mean Monthly Temperature) | | | 0.13 | -12.56 | 12.82 |
| Percent Aquatic Agriculture 1km Radius | | | 1.05 | -0.99 | 3.09 |
| *Note:* | |  | |  | *p<0.05 |

Correlation test output of larval variables:

> cor.test(df2_m$percent_rf_1km,df2_m$sm_avg)

Pearson's product-moment correlation

data: df2_m$percent_rf_1km and df2_m$sm_avg

t = -2.0509, df = 151, p-value = 0.042

alternative hypothesis: true correlation is not equal to 0

95 percent confidence interval:

-0.315072783 -0.006106502

sample estimates:

cor

-0.1646251

> cor.test(df2_m$ndvi,df2_m$percent_forest_2km)

Pearson's product-moment correlation

data: df2_m$ndvi and df2_m$percent_forest_2km

t = 1.409, df = 151, p-value = 0.1609

alternative hypothesis: true correlation is not equal to 0

95 percent confidence interval:

-0.04558186 0.26775820

sample estimates:

cor

0.1139202

> cor.test(df2_m$prec,df2_m$sm_avg)

Pearson's product-moment correlation

data: df2_m$prec and df2_m$sm_avg

t = 10.904, df = 151, p-value < 2.2e-16

alternative hypothesis: true correlation is not equal to 0

95 percent confidence interval:

0.5644848 0.7440322

sample estimates:

cor

0.6637132

> cor.test(df2_m$percent_rf_1km,df2_m$sm_avg)

Pearson's product-moment correlation

data: df2_m$percent_rf_1km and df2_m$sm_avg

t = -2.0509, df = 151, p-value = 0.042

alternative hypothesis: true correlation is not equal to 0

95 percent confidence interval:

-0.315072783 -0.006106502

sample estimates:

cor

-0.1646251

| **MEAN MONTHLY TEMPERATURE (DEGREES C)** | | | | | | | | | | | | | | | | |  | |  | |  | |
| --- | --- | --- | --- | --- | --- | --- | --- | --- | --- | --- | --- | --- | --- | --- | --- | --- | --- | --- | --- | --- | --- | --- |
|  | SE.1 | SE.2 | SE.3 | SE.5 | SE.6 | SW.3 | SW.5 | WC.2 | WC.3 | WC.6 | HP.1 | HP.2 | HP.3 | HP.4 | HP.6 |  | |  | |  | |  |
| January | 26 | 26.1 | 26.2 | 26.1 | 26 | 27.8 | 27.9 | 27.8 | 27.9 | 27.8 | 19.7 | 20.1 | 19.9 | 21.6 | 20 |  | |  | |  | |  |
| February | 26 | 25.9 | 26.1 | 25.9 | 25.9 | 27.3 | 27.3 | 27.8 | 27.9 | 27.8 | 19.6 | 20 | 19.7 | 21.4 | 19.9 |  | |  | |  | |  |
| March | 25.4 | 25.5 | 25.6 | 25.5 | 25.4 | 26.6 | 26.6 | 27.2 | 27.2 | 27 | 19.1 | 19.5 | 19.3 | 21 | 19.4 |  | |  | |  | |  |
| April | 24.3 | 24.4 | 24.5 | 24.3 | 24.3 | 25.1 | 25 | 25.7 | 25.8 | 25.7 | 18 | 18.3 | 18.1 | 19.9 | 18 |  | |  | |  | |  |
| May | 22.4 | 22.4 | 22.4 | 22.2 | 22 | 22.6 | 22.6 | 23.3 | 23.3 | 23.3 | 15.6 | 16 | 15.7 | 17.5 | 15.7 |  | |  | |  | |  |
| June | 20.6 | 20.7 | 20.6 | 20.6 | 20.4 | 21 | 21.1 | 21.5 | 21.4 | 21.3 | 13.7 | 14.1 | 13.8 | 15.7 | 13.8 |  | |  | |  | |  |
| July | 19.9 | 19.9 | 20 | 19.7 | 19.6 | 20.2 | 20.2 | 20.5 | 20.5 | 20.3 | 12.9 | 13.1 | 12.9 | 14.9 | 13 |  | |  | |  | |  |
| August | 19.9 | 20 | 20.1 | 20 | 19.8 | 20.8 | 20.7 | 21.6 | 21.6 | 21.5 | 13.2 | 13.6 | 13.2 | 15.7 | 13.5 |  | |  | |  | |  |
| September | 20.8 | 20.9 | 21 | 20.7 | 20.7 | 22.1 | 22.2 | 22.8 | 22.7 | 23 | 14.8 | 15.1 | 15 | 17.5 | 15.3 |  | |  | |  | |  |
| October | 22.5 | 22.5 | 22.5 | 22.3 | 22.5 | 23.9 | 23.8 | 24.7 | 24.7 | 24.8 | 17.3 | 17.8 | 17.5 | 19.9 | 17.8 |  | |  | |  | |  |
| November | 24.3 | 24.3 | 24.3 | 24.2 | 24.3 | 25.9 | 25.8 | 26.5 | 26.3 | 26.5 | 19 | 19.4 | 19.2 | 21.2 | 19.2 |  | |  | |  | |  |
| December | 25.4 | 25.4 | 25.4 | 25.3 | 25.3 | 27.2 | 27.2 | 26.9 | 26.9 | 27 | 19.6 | 19.9 | 19.7 | 21.5 | 19.7 |  | |  | |  | |  |
|  |  |  |  |  |  |  |  |  |  |  |  |  |  |  |  |  | | Month of sampling | | | |  |
| **TOTAL MONTHLY PRECIPITATION (MM)** | | | | | | | | | | | | | | | | |  | | Month before sampling | | | |
|  | SE.1 | SE.2 | SE.3 | SE.5 | SE.6 | SW.3 | SW.5 | WC.2 | WC.3 | WC.6 | HP.1 | HP.2 | HP.3 | HP.4 | HP.6 |  | |  | |  | |  |
| January | 32.2 | 33.2 | 33.5 | 33 | 34.8 | 7.8 | 7.7 | 14.1 | 13.6 | 15.5 | 29.2 | 29.8 | 29.4 | 31.5 | 29 |  | |  | |  | |  |
| February | 32.7 | 37 | 36.1 | 34.3 | 35.7 | 8 | 8.4 | 16.6 | 16.4 | 16.9 | 24.3 | 24.7 | 24.4 | 24.7 | 23.9 |  | |  | |  | |  |
| March | 37.1 | 36.4 | 36.8 | 38 | 38.7 | 3.2 | 3.6 | 6.5 | 6.3 | 7.9 | 22.6 | 23.3 | 22.9 | 22.3 | 20.9 |  | |  | |  | |  |
| April | 18.6 | 22.5 | 20.8 | 19.1 | 16.7 | 1.2 | 1 | 0.7 | 0.7 | 1.2 | 8.5 | 8.9 | 8.5 | 6.9 | 7.9 |  | |  | |  | |  |
| May | 17.5 | 19.7 | 18.1 | 16.7 | 12.9 | 1.6 | 1.7 | 0.5 | 0.5 | 0.6 | 4.5 | 4.6 | 4.6 | 2.6 | 3.5 |  | |  | |  | |  |
| June | 16.8 | 17.6 | 16.6 | 16.3 | 13.3 | 0.9 | 1 | 0.2 | 0.2 | 0.3 | 3.8 | 3.9 | 3.9 | 1.7 | 2.4 |  | |  | |  | |  |
| July | 14.1 | 15.1 | 14.3 | 14.2 | 12.5 | 0.4 | 0.4 | 0 | 0 | 0.1 | 3.4 | 3.5 | 3.5 | 1.5 | 2.2 |  | |  | |  | |  |
| August | 13.6 | 13.8 | 13.4 | 13.6 | 12.5 | 0.4 | 0.4 | 0.2 | 0.2 | 0.2 | 3.2 | 3.3 | 3.3 | 1.5 | 2.1 |  | |  | |  | |  |
| September | 9 | 9.7 | 9.4 | 9.2 | 8.6 | 0.9 | 0.8 | 0.3 | 0.3 | 0.4 | 3.6 | 3.7 | 3.7 | 2.6 | 3.1 |  | |  | |  | |  |
| October | 7.9 | 8.8 | 8.4 | 8.2 | 7.7 | 1.6 | 1.5 | 1 | 1 | 1.5 | 5.3 | 5.3 | 5.2 | 5.6 | 5.8 |  | |  | |  | |  |
| November | 16.2 | 15.5 | 15.8 | 16.2 | 16.8 | 3.3 | 3.2 | 2.9 | 2.9 | 4.1 | 16.7 | 17 | 16.8 | 15.8 | 16.4 |  | |  | |  | |  |
| December | 21.8 | 22.5 | 22.4 | 21.4 | 21.2 | 5.7 | 5.5 | 9.3 | 9.1 | 11.2 | 26.7 | 27.1 | 26.8 | 27.7 | 26.6 |  | |  | |  | |  |

**Table 2.** Temperature and precipitation information for month of larval sampling and preceding month.
